# Effects of Coenzyme Q10 Supplementation on Physical Function Adaptations to High-Intensity Interval Training in Older Adults

**DOI:** 10.3390/nu17243959

**Published:** 2025-12-18

**Authors:** Navid Bagheri, Mehdi Kargarfard, Reza Bagheri, Frédéric Dutheil

**Affiliations:** 1 Department of Exercise Physiology, Faculty of Sport Sciences, University of Isfahan, Isfahan 81746-73441, Iran; navid.bagheri@spr.ui.ac.ir (N.B.); will.fivb@yahoo.com (R.B.); 2 Université Clermont Auvergne, CNRS, LaPSCo, Physiological and Psychosocial Stress, CHU Clermont-Ferrand, University Hospital of Clermont-Ferrand, Preventive and Occupational Medicine, Witty Fit, F-63000 Clermont-Ferrand, France; frederic.dutheil@uca.fr

**Keywords:** coenzyme Q10, elderly, high-intensity interval training, physical function, exercise adaptation, dietary supplements

## Abstract

**Objectives:** This study investigated whether CoQ10 supplementation enhances physical adaptations to high-intensity interval training (HIIT) in muscular strength, power, and physical function in older adults. **Method:** In a double-blind, randomized controlled trial, 38 adults aged 65–75 were assigned to either a CoQ10 (Females: 8; Males: 11) or placebo (Females: 8; Males: 11) group and completed an 8-week supervised HIIT program. Lower- and upper-body strength (30s 5-repetition chair stand [5XSST], chair standing [30CST], handgrip strength [HGR/L]), balance (single-leg stand [SLS], timed up and go [TUG]), mobility (25-foot walk [25FW]), and aerobic endurance (6-minute walk [6MWT]) were assessed pre- and post-intervention. **Results:** The CoQ10 group demonstrated significantly greater improvements in 5XSST and 30CST compared to the placebo group (*p* < 0.05). Both groups showed significant within-group improvements in right and left handgrip strength, SLS, 6MWT, and TUG (all *p* < 0.001), with no significant between-group differences observed for these outcomes (*p* > 0.05). No adverse events were reported. **Conclusion:** While CoQ10 supplementation enhanced improvements in lower-body strength and power, as indicated by the greater gains in 5XSST and 30CST performance compared to the placebo, no between-group differences were observed in TUG, grip strength, or other functional outcomes. This suggests that the performance-related effects of CoQ10 may be more specific to muscular power output and fatigue resistance, rather than general mobility or balance-related tasks. These findings highlight the potential of CoQ10 as a targeted adjunct in exercise for supporting lower-body function and physical performance in older adults.

## 1. Introduction

Aging is associated with a gradual decline in the function of various cells and tissues, impacting multiple systems such as the musculoskeletal and cardiorespiratory systems, and consequently reducing quality of life [1,2]. With age, body fat distribution shifts and accumulates, heightening the risk of metabolic diseases [3]. Among the most notable changes are physiological alterations and shifts in body composition, where muscle mass decreases and fat tissue increases, even when body mass remains stable [4,5]. These changes, coupled with declines in cardiovascular and respiratory function, contribute to decreased physical function [6,7]. As a result, older adults often experience increased fatigue and a higher risk of falls during daily activities, further diminishing their overall quality of life [8,9,10].

Exercise interventions are widely recommended to mitigate the effects of aging and improve physical function [11,12,13]. Regular exercise has been shown to preserve muscle mass, reduce fat accumulation, and maintain physical performance [14,15,16,17]. Among the various forms of exercise, high-intensity interval training (HIIT) has emerged as an efficient method for improving aerobic endurance, strength, and power [18,19,20]. The benefits of HIIT are attributed to improvements in body composition and cellular adaptations, including enhanced organelle function [21,22]. Although HIIT can be performed using different exercise modalities, cycling was selected in the present study because it offers greater stability, lower joint impact, and improved safety for older adults compared with running or other weight-bearing activities [20]. Furthermore, cycling-based endurance training appears to support the preservation of muscle mass, strength, and power in aging adults [23,24,25].

Nutritional supplements also play a significant role in influencing physical performance and body composition. Research has explored the effects of supplements on aging-related changes in older adults [26,27], with evidence suggesting that combining exercise with nutrition is more effective than either intervention alone [28]. Several studies have examined age-related changes in nutrient intake and metabolism, exploring their potential role in supporting body composition and physical function in older adults [29,30,31].

One such nutrient is Coenzyme Q10 (CoQ10), a naturally occurring antioxidant essential for energy production within cells. CoQ10 levels tend to decline with aging, potentially leading to reduced mitochondrial activity, increased oxidative stress, and impaired physical function [32,33,34,35]. This decline in CoQ10 has been linked to decreased muscle strength, endurance, and overall fatigue [36]. While some studies have reported no significant effects of CoQ10 supplementation on exercise performance [37,38], others have suggested potential benefits [39,40,41].

Despite the growing body of research on CoQ10 and exercise, few studies have investigated the combined effects of CoQ10 supplementation and HIIT on physical performance in older adults. This study aims to address this gap by evaluating whether an eight-week HIIT program supplemented with CoQ10 can lead to improvements in physical function in older adults. We hypothesized that CoQ10 supplementation would enhance HIIT-induced gains in strength, mobility, and endurance in older adults.

## 2. Methods

### 2.1. Participants

This study enrolled 38 healthy older adults (22 men and 16 women), aged 65 to 75 years, who met predefined inclusion criteria. Participants were considered sedentary if they had engaged in less than one hour of structured physical activity per week over the previous year. Recruitment was conducted via social media platforms, where interested individuals were invited to contact the research team. Those who responded received detailed information about the study objectives and procedures either over the phone or through in-person meetings held in local parks, depending on their preference.

To assess eligibility, all participants completed a comprehensive health and fitness questionnaire covering recent physical activity levels (past six months), sleep duration, and current use of dietary supplements or medications. Inclusion criteria required participants to be free from cardiovascular diseases and orthopedic disorders that could interfere with physical activity. Only individuals who met these criteria and provided written and verbal informed consent were included in the study.

This study was conducted according to the guidelines laid down in the Declaration of Helsinki, and all procedures involving human subjects were approved by the university’s ethics committee; IR.UI.REC.1403.018; approval date: 9 March 2024 and registered with the Iranian Registry of Clinical Trials; IRCT20240310061243N1; approval date: 25 July 2024 https://irct.behdasht.gov.ir/ (accessed on 25 July 2024). Written and verbal informed consent was obtained from all subjects.

### 2.2. Body Composition

Body composition was evaluated using a bioelectrical impedance analysis (BIA) device (model: Inbody126, InBody, Seoul, Republic of Korea). Measurements were performed in the morning after an overnight fast to minimize variability due to recent food or fluid intake. Participants were instructed to avoid strenuous physical activity and alcohol for 24 h prior to testing. Standardized procedures were followed, with participants standing barefoot with minimal clothing on the device and maintaining a neutral posture. Parameters assessed included body fat mass, skeletal muscle mass, and total body weight. All assessments were conducted by trained personnel to ensure accuracy and consistency.

### 2.3. Study Design

**Figure 1:**
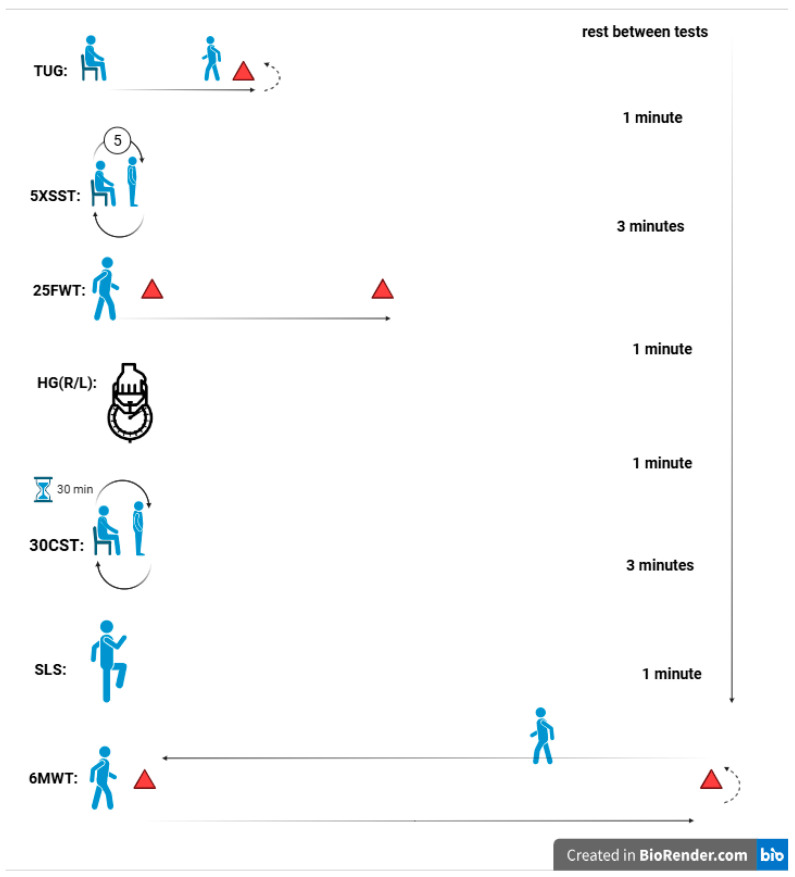
Schematic design of the study.

This study employed an experimental, randomized, double-blinded design with baseline and post-intervention measurements. Participants were randomly assigned to two equal-sized groups: one underwent eight weeks of HIIT combined with CoQ10 supplementation (Females: 8; Males: 11), while the other received HIIT with a placebo (Females: 8; Males: 11). Participants first completed two days of preliminary testing. On the first day, blood pressure was assessed, and questionnaires were administered. The second day involved physical function tests, both before the intervention (baseline) and after the final intervention session (at 8 weeks). Randomization was stratified by gender (male/female) to ensure balanced group composition. The schematic design of the study is shown in Figure 1.

### 2.4. Intervention

#### 2.4.1. HIIT Protocol

The low-volume HIIT protocol was implemented in accordance with the guidelines previously established [42]. The intervention was conducted three times per week over an 8-week period. Participants performed the low-volume HIIT sessions using electronically braked cycle ergometers. Each session began with a 2-minute warm-up, followed by five 1-minute HIIT performed at intensities ranging from 80% to 95% of the individual’s peak heart rate, interspersed with 1-minute low-intensity recovery periods. Sessions concluded with a 3-minute cool-down, resulting in a total session duration of 14 min. The minimum exercise intensity, measured as heart rate, was progressively increased over the 8-week period as follows: 80–85% of peak heart rate during weeks 1–3, 85–90% during weeks 4–6, and 90–95% during weeks 7–8. Training sessions were scheduled to take place between 7:30 a.m. and 10:30 a.m. It should be noted that before each training session, resting blood pressure was assessed using a sphygmomanometer, and participants were screened for any cardiovascular risks that might contraindicate high-intensity exercise. Additionally, several recent studies have examined comparable high-intensity training loads in older adults and have reported good feasibility and safety of such protocols [43,44].

#### 2.4.2. CoQ10 Supplementation

In the experimental group, participants took CoQ10 soft gels daily with lunch at a prescribed dosage of 100 mg, a dose shown in previous clinical studies to be safe and effective in improving markers of oxidative stress and exercise-induced muscle damage in adults [45,46]. The supplement was obtained from Vana Darou Gostar Company (Isfahan, Iran), a reputable manufacturer in Iran, and is commercially available as a single-ingredient CoQ10 preparation. Since HIIT sessions were scheduled in the morning, supplementation effectively occurred after exercise on training days. To ensure adherence, participants received text message reminders about their scheduled intake. At the end of the study, they were asked to return the packaging, including any leftover tablets. The placebo was manufactured using inactive ingredients designed to closely resemble the appearance of CoQ10 soft gels. These ingredients matched the color, shape, and size of the actual supplement. Additionally, the placebo was packaged and labeled identically to the CoQ10 supplement.

### 2.5. Outcome Measures

#### 2.5.1. Physical Function Tests

Functional tests were conducted at approximately 10:00 a.m. to ensure participants had consumed their morning meal and were not experiencing fatigue. To optimize physical and mental readiness, a standardized warm-up routine was implemented before testing. This routine included one minute of walking at a comfortable pace, followed by shoulder rolls (1 set of 8 repetitions), leg swings (1 set of 8 repetitions), mini squats (1 set of 5 repetitions), and calf raises (1 set of 8 repetitions). Functional tests were performed in a predetermined sequence, with rest intervals of 1 to 3 min between each test to ensure consistency.

#### 2.5.2. 5-Repetition Chair Stand Test (5XSST)

The 5-Repetition Chair Stand Test was employed to assess lower-body power and functional capacity. A stopwatch was used to measure the time it took for the participant to complete all five chair stands [47]. Participants were instructed to rise from a seated position to a full standing position and return to a seated position for a total of five repetitions. A standard chair with a seat height of 43 cm was used to ensure consistency across all participants. The time taken to complete all five repetitions was recorded using a stopwatch. To prevent the use of their hands for assistance, participants were instructed to cross their arms over their chest and perform the test as quickly as possible while maintaining proper form and safety. This test primarily reflects muscle power and the ability to perform functional movements.

#### 2.5.3. Thirty-Second Chair Stand Test (30CST)

The 30-Second Chair Stand Test was used to assess lower body strength and muscular endurance. The procedure was identical to the 5-Repetition Chair Stand Test, except that participants were instructed to complete as many full stands as possible within 30 s. The total number of repetitions performed was recorded, providing a measure of lower limb strength. These tests serve as reliable indicators of functional capacity, reflecting the participants’ ability to perform daily activities [48]. This test complements the 5XSST by providing a measure of muscular strength and endurance over a fixed time period, capturing a different aspect of lower-limb functional performance.

#### 2.5.4. Handgrip Test

Grip strength was measured using a Saehan Hand Dynamometer (Model: SH5000-3, Saehan Corporation, Masan, Republic of Korea). Participants stood in an upright position, holding the dynamometer in one hand with their arm fully extended at their side. While maintaining this posture, they were instructed to squeeze the dynamometer as hard as possible for about 3–5 s [49].

#### 2.5.5. Timed Up and Go Test (TUG)

To assess dynamic balance and the risk of falls, the Timed Up and Go (TUG) test was used. Participants began by sitting in a chair with their backs against the backrest and their arms resting comfortably. Upon hearing the cue “go,” they rose from the chair, walked 3 m to a designated point, turned around, walked back to the chair, and sat down again (Figure 1). Timing started as soon as the participant initiated the motion to rise from the chair [50].

#### 2.5.6. 25-Foot Walk Test (25FW)

The 25-Foot Walk Test was used to assess walking speed and mobility. Participants were timed as they walked a straight 25-foot distance on a flat, unobstructed path to evaluate leg function and mobility [51]. Each participant started from a standing position behind the designated starting line and was instructed to walk as quickly and safely as possible to the 25-foot mark (Figure 1). Timing began when the first foot left the starting line and stopped when the foot crossed the finish line. The test was conducted twice, with a brief rest period between trials to minimize fatigue. The fastest time recorded from the two trials was used for analysis, as it provides a reliable measure of optimal walking speed and mobility. Prior to testing, proper instructions and demonstrations were given, and participants were closely monitored to ensure safety throughout the test.

#### 2.5.7. Single-Leg Stand Test (SLS)

Balance and stability were assessed using a one-leg stand test, which measures the duration a participant can maintain balance on one leg. The test was performed with eyes open, and Participants were instructed to stand on one leg with the opposite leg bent at the knee, ensuring the raised foot did not touch the supporting leg. Arms were kept at the sides, and participants were asked to focus on a fixed point to aid stability (Figure 1). The test was conducted on a flat, non-slip surface to ensure safety. Timing began when the participant lifted one foot off the ground and ended when they lost balance, as indicated by touching the ground with the raised foot, using their arms for support, or shifting the supporting foot. The time participants maintained this position was recorded as an objective measure of balance and stability. Each participant completed two trials per leg to ensure reliability and consistency. The longer duration from the two attempts was recorded as the final measure of balance for that leg [52].

#### 2.5.8. Six Minute Walk Test (6MWT)

The 6-Minute Walk Test was used to assess functional exercise capacity, endurance, and mobility. A straight, level 30-meter walkway with clear markings at both ends was designated as the testing area to ensure consistency. Before the test, participants received detailed instructions and were encouraged to walk back and forth along the designated path at a comfortable yet sustainable pace for the entire six-minute duration. The goal was to cover the maximum distance possible without running or jogging. Timing began when participants started walking and ended precisely at the six-minute mark [53].

#### 2.5.9. Dietary Intake and Physical Activity Evaluation

Assessment of energy and macronutrient consumption was conducted using a 24 h recall questionnaire over a span of three days, covering one weekend day and two nonconsecutive weekdays at the beginning of the study. The International Physical Activity Questionnaire—Short Form was utilized to gauge the extent of daily physical activity [54]. Then, the intake of each macronutrient was estimated with Caltivita software version 2.1.0 (2103). Data are shown in Table 1. Statistical analysis revealed no significant differences in fat consumption between the experimental and control groups at either time point. (P_Pre_ = 0.746, P_Post_ = 0.901) The percentage of total caloric intake derived from fat remained stable throughout the study. Given the lipophilic nature of CoQ10, the consistency in dietary fat intake across time and between groups was considered important to ensure comparable absorption of the supplement [55,56]. Participants in this study were physically and mentally healthy and did not report the use of high-dose medications known to interfere with Coenzyme Q10 metabolism [57]. Moreover, none of the individuals reported symptoms typically associated with CoQ10 deficiency due to medication use, such as fatigue syndrome [58].

### 2.6. Statistical Analysis

The required sample size for this study was determined using G*Power software (version 3.1.9.2). The calculation was based on a statistical power of 0.80 (1-β), an alpha level of 0.05, and a large effect size (f(V) = 0.25). Based on these parameters, a minimum of 34 participants was considered sufficient. A repeated-measures ANOVA design with a within-between interaction was employed, incorporating two groups (CoQ10 + HIIT vs. placebo + HIIT), two measurement time points (pre and post), and gender (male vs. female) as an additional between-subjects factor. The normality of the data was assessed using the Shapiro–Wilk test. Levene’s test was used to assess homogeneity of variances, and Box’s M test was applied to test equality of covariance matrices. A three-way mixed ANOVA (Time × Group × Gender) was performed to assess the effects of the intervention. When significant interactions or main effects were observed, post hoc analyses were conducted using Tukey’s test. A significance level of *p* < 0.05 was set for all statistical analyses. Although multiple functional outcomes were analyzed as co-primary endpoints, no formal correction for multiple testing was applied. Results are interpreted cautiously, acknowledging the potential for increased type I error.

## 3. Results

### 3.1. Participant Characteristics

**Figure 2:**
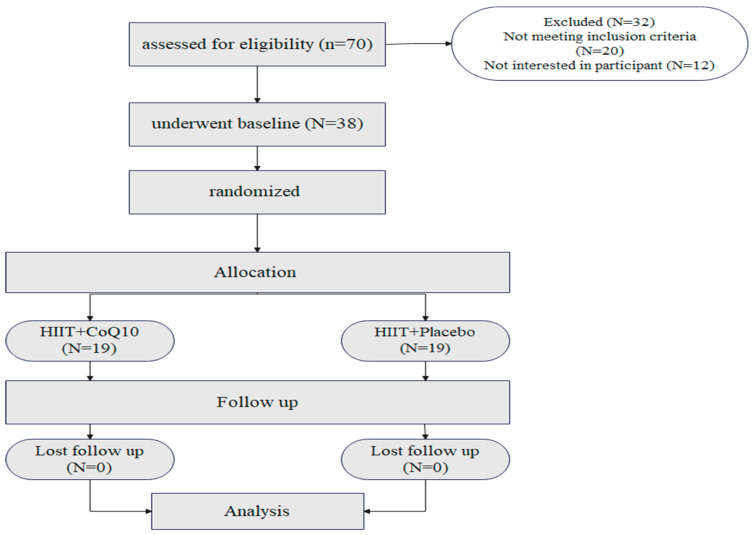
Flow of the participant recruitment.

Seventy participants were assessed for eligibility. Twenty did not meet the inclusion criteria, while 12 were not interested in participating after the first interview (Figure 2). All participants completed the study with no loss to follow-up. Both groups had similar ages, sex, and BMI. Baseline characteristics of participants are shown in Table 1. No adverse events occurred or were reported during the intervention period.

### 3.2. Adherence and Fidelity

Adherence was assessed by counting returned capsules each week. Compliance was high in both groups, and the adherence percentages are presented in Table 2.

### 3.3. Physical Function

**Figure 3:**
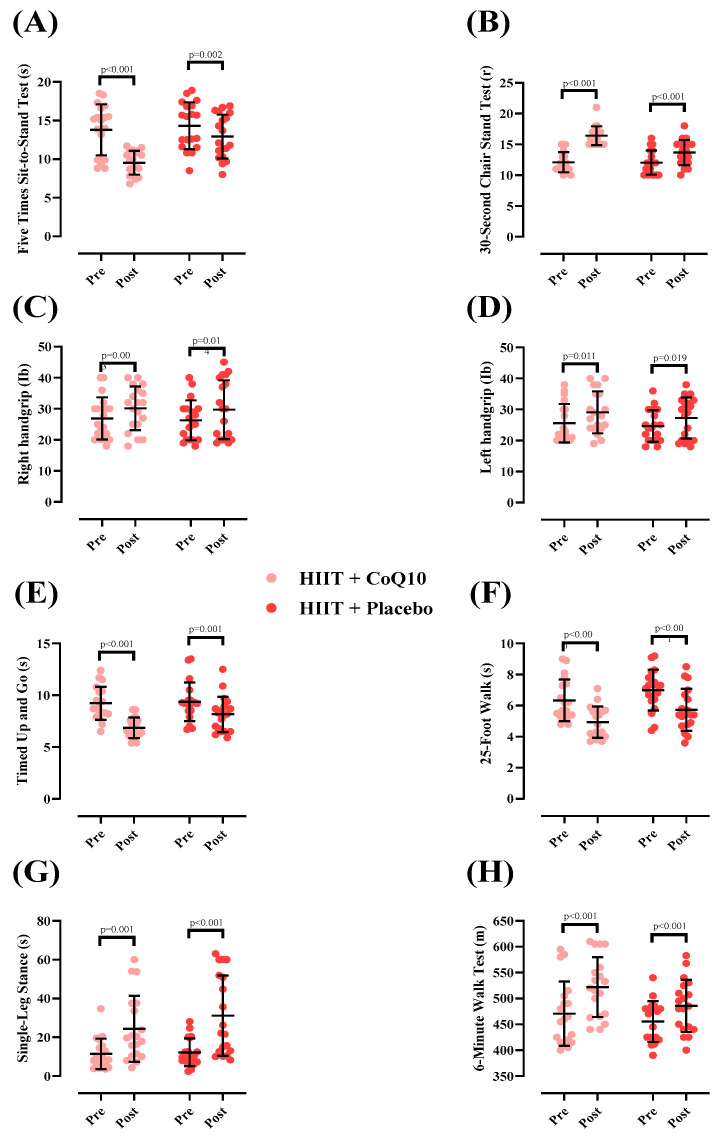
Changes in physical function throughout the intervention. *N* = 19 per group; error bars represent standard deviation (SD). (**A**) 5-Times Sit-to-Stand Test (s); (**B**) 30-Second Chair Stand Test(r); (**C**) Right hand grip (Ib); (**D**) Left hand grip (Ib); (**E**) Timed up and go test (s); (**F**) 25-Foot Walk test (s); (**G**) Single-Leg Stand (s); (**H**) Six Minute Walk Test (m).

Measures of physical function at pre- and post-intervention and between groups are shown in Figure 3. There was a significant group-by-time interaction for 5XSST [*p* < 0.001; (Figure 3A)] The 5XSST time significantly decreased in HIIT + CoQ10 [−4.26 s (95% CI: −5.73 to −2.79), *p* < 0.001] and HIIT + P groups [−1.40 s (95% CI: −2.19 to −0.60), *p* = 0.002]. The decrease in 5XSST in HIIT + CoQ10 was significantly greater than in HIIT + placebo (F = 5.92); (η^2^_p_ = 0.141); (*p* = 0.020).

There was a significant group-by-time interaction for 30CST [*p* < 0.001; (Figure 3B)]. The 30CST repetitions significantly increased in HIIT+CoQ10 [4.31 repetitions (95% CI: 3.46 to 5.16), *p* < 0.001] and HIIT + P groups [1.63 repetitions (95% CI: 1.17 to 2.09), *p* < 0.001]. The increase in 30CST in HIIT + CoQ10 was significantly greater than in HIIT + placebo [(F= 6.68); (η^2^_p_ = 0.156); (*p* = 0.020).

For right-handgrip strength, a significant increase was observed in the HIIT + CoQ10 group [3.26 Ib, (95% CI: 1.27 to 5.24), *p* = 0.003] and HIIT + P group [3.42 Ib, (95% CI: 0.78 to 6.05), *p =* 0.014] groups, with no between-group differences (F = 0.057); (η^2^_p_ = 0.002); (*p* = 0.812); (Figure 3C).

For left-handgrip strength, there was a significant increase in the HIIT + CoQ10 [3.52 Ib, 95% CI: 0.89 to 6.15), *p* = 0.011] and the HIIT + P groups [2.57 Ib, 95% CI: 0.48 to 4.67), *p* = 0.019] with no between-group differences (F = 0.550); (η^2^_p_ = 0.015); (*p* = 0.463); (Figure 3D).

The TUG time significantly decreased in HIIT + CoQ10 [−2.37 s (95% CI: −3.00 to −1.74), *p* < 0.001] and HIIT + P groups [−1.20 s (95% CI: −1.79 to −0.60), *p* = 0.001] with no between-group differences (F = 2.34); (η^2^_p_ = 0.061); (*p* = 0.134); (Figure 3E).

The 25FW time significantly decreased in HIIT + CoQ10 [−1.45 s (95% CI: −1.95 to −0.94), *p* < 0.001] and HIIT + P groups [−1.26 s (95% CI: −1.87 to −0.66), *p* < 0.001]. However, there was no significant difference between groups (F = 4.24); (η^2^_p_ = 0.105) (*p* = 0.056); Figure 3F).

The SLS duration significantly increased in HIIT + CoQ10 [12.92 s (95% CI: 5.78 to 20.06), *p* = 0.001] and HIIT + P groups [18.93 s (95% CI: 10.61 to 27.25), *p* < 0.001], with no significant difference between groups (F = 0.948); (η^2^_p_ = 0.026); (*p* = 0.337); (Figure 3G).

The 6MWT distance significantly increased in HIIT + CoQ10 [51.26 m (95% CI: 39.62 to 62.89), *p* < 0.001] and HIIT+P groups [30.31 m (95% CI: 18.92 to 41.70), *p* < 0.001], with no significant difference between groups after the intervention (F = 2.37); (η^2^_p_ = 0.062); (*p* = 0.132); (Figure 3H).

A three-way mixed ANOVA revealed no significant Time × Group × Gender interaction effects for any of the measured variables (*p* > 0.05), indicating that the intervention effects did not differ between males and females across the groups over time (see Table 3).

The SMM significantly increased in HIIT+CoQ10 [0.68 kg (95% CI: 0.36 to 1.01), *p* < 0.001] but not in HIIT+P group [0.48 kg (95% CI: −0.01 to 0.98), *p* = 0.058], with no significant difference between groups after the intervention (F = 0.017); (η^2^_p_ = 0.000); (*p* = 0.894).

**Figure 4:**
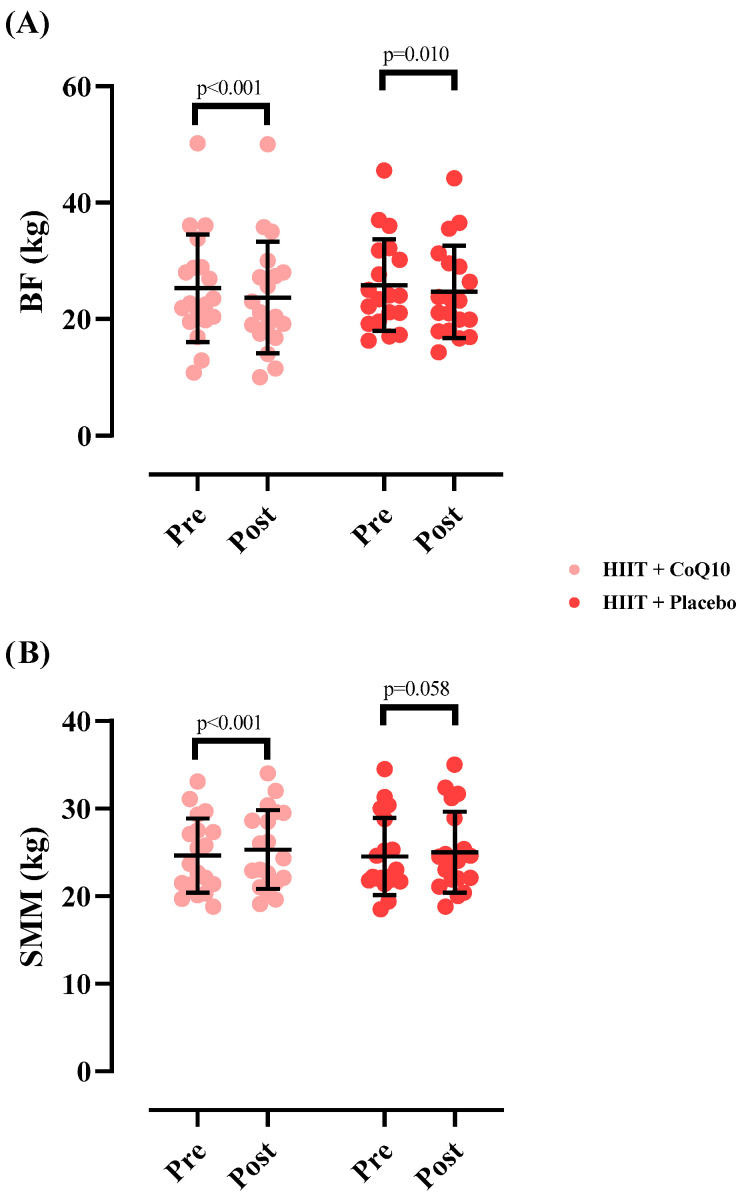
Changes in skeletal muscle mass (**A**) and fat mass (**B**) throughout the intervention. *N* = 19 per group, error bars represent standard deviation (SD).

Finally, the BF significantly decreased in HIIT+CoQ10 [−1.56 kg (95% CI: 2.22 to 0.90), *p* < 0.001] but not in HIIT+P group [−1.15 kg (95% CI: −1.99 to 0.30), *p* = 0.010], with no significant difference between groups after the intervention (F = 0.071); (η^2^_p_ = 0.002); (*p* = 0.790). Figure 4 illustrates the changes in body composition measures across the intervention period.

### 3.4. Dietary Assessments

Average dietary intakes at baseline and throughout the intervention are presented in Table 4. No significant differences were observed in total energy intake (P_Pre_ = 0.744, P_Post_ = 0.770) or in macronutrients, including carbohydrates (P_Pre_ = 0.842, P_Post_ = 0.838), protein (P_Pre_ = 0.775, P_Post_ = 0.705), and fat (P_Pre_ = 0.746, P_Post_ = 0.901).

## 4. Discussion

This study examined whether eight weeks of CoQ10 supplementation could enhance physical performance adaptations to high-intensity interval training (HIIT) in 38 healthy older adults. HIIT improved several domains of physical function, including muscular strength and power (30CST, 5XSST), gait and balance (TUG, SLS, 25FW), and cardiovascular endurance (6MWT).

Notably, participants in the HIIT + CoQ10 group showed significantly greater improvements in lower-body strength and power, particularly in 5XSST and 30CST outcomes. The reduced completion time in 5XSST and increased 30CST repetitions suggest enhanced lower-body muscular strength and functional power. These benefits may be partly related to the potential effects of CoQ10’s role in reducing muscular fatigue and improving energy metabolism [59,60]. Age-related declines in mitochondrial efficiency and increased reactive oxygen species (ROS) contribute to fatigue during repeated high-effort tasks like 5XSST and 30CST [61,62]. CoQ10 is hypothesized to support mitochondrial electron transport, enhancing ATP production while reducing ROS accumulation [41]. It also activates the Nrf2 pathway, upregulating antioxidant enzymes such as SOD, CAT, and GPx [63,64]. These enzymes help neutralize excessive ROS, protecting muscle fibers and delaying fatigue onset [65,66]. Together, these mechanisms may contribute to the improved muscular endurance and power seen in the CoQ10 group, particularly relevant for older adults prone to oxidative damage and mitochondrial dysfunction [67]. Performance gains were consistent across sexes, as the Time × Group × Gender interaction was not significant.

In contrast, handgrip strength improved in both groups, but no between-group differences emerged post-intervention. This suggests CoQ10’s benefits may be more relevant to dynamic, repetitive lower-body movements than to static upper-limb strength tasks [68]. Such differences may reflect variations in mitochondrial density, perfusion, and fiber-type distribution between upper and lower limbs, which influence adaptation to oxidative stress [69]. The improvement observed in both groups likely reflects the systemic effects of HIIT, as high-intensity aerobic exercise can reduce systemic inflammation and enhance overall neuromuscular function, leading to gains even in non-trained muscle groups such as the upper limb [70,71].

Body composition changes followed a similar pattern in both groups. Although SMM increased and BF decreased significantly within each group, no between-group differences emerged. These findings suggest that the superior gains in lower-body strength and power observed in the CoQ10 group were not attributable to differential changes in muscle mass or fat mass. These results align with previous research indicating that HIIT effectively improves body composition and functional fitness in older adults [72,73]. Notably, no significant differences were observed between the HIIT+CoQ10 and HIIT + placebo groups, suggesting that CoQ10 supplementation did not provide additional benefits beyond those achieved by HIIT alone.

CoQ10 and HIIT may interact via ROS-mediated adaptive signaling pathways. During intense cycling, transient ROS elevations trigger beneficial signaling pathways, despite their potential for harm at higher levels. This includes Nrf2 activation, which upregulates antioxidant defenses like SOD, CAT, and GPx [63,64]. ROS also stimulate PGC-1α expression, promoting mitochondrial biogenesis and improved muscle oxidative capacity [74]. CoQ10 may reinforce these training-induced adaptations by optimizing redox balance and amplifying Nrf2 activation [63]. Collectively, these mechanisms may help reduce fatigue and support improvements in muscular strength, endurance, and recovery.

Despite gains in lower-body strength and power, CoQ10 had no added effects on balance or gait, as shown by unchanged TUG, 25FW, and SLS outcomes between groups. This suggests CoQ10’s ergogenic benefits may be limited to power and fatigue resistance, rather than general mobility or balance. This interpretation is consistent with previous studies suggesting that CoQ10’s primary benefits lie in enhancing muscle function, particularly in exercises requiring strength and endurance [33,75].

The absence of between-group differences in 6MWT reinforces the specificity of CoQ10’s effects to strength and power, not endurance. Notably, CoQ10 was consumed after exercise sessions, which may have limited its acute ergogenic effects during HIIT. Administering the supplement pre-exercise might enhance mitochondrial energy production and antioxidant support during the workout, potentially amplifying its benefits. Both groups improved in walking distance, likely due to HIIT alone, which may have masked any subtle effects of CoQ10 on endurance. CoQ10’s impact on endurance may be more evident in individuals with lower baseline function or statin-induced fatigue [76].

These findings are consistent with prior research showing CoQ10 is most effective during high-effort, strength-based muscle tasks [77]. The lack of additional benefits in endurance or mobility may reflect ceiling effects or the strong adaptations induced by HIIT alone. The interpretation of the present findings should be considered in light of the absence of baseline serum CoQ10 measurements, which limit definitive conclusions regarding the specific contribution of supplementation. Although accumulating evidence indicates that circulating CoQ10 levels are associated with physical performance and muscle function in older adults [33], functional measures cannot be used as a surrogate for biochemical status. Accordingly, despite the absence of significant between-group differences in physical performance at baseline, meaningful interindividual variability in endogenous CoQ10 levels cannot be excluded, and the observed findings should therefore be interpreted with appropriate caution.

## 5. Limitations

This study has several limitations. First, although the study was able to detect moderate effects, the sample size was insufficient to permit robust subgroup analyses based on factors such as baseline fitness, comorbidities, and sex-specific responses. Second, the reliance on self-reported dietary intake and habitual physical activity may have introduced recall and reporting bias, as no objective monitoring was implemented outside of supervised training sessions. Third, plasma CoQ10 concentrations were not measured at baseline or post-intervention, which precluded biochemical confirmation of individual absorption, meaningful interindividual variability in endogenous CoQ10 status, and direct associations between circulating CoQ10 levels and changes in functional performance outcomes. Fourth, CoQ10 supplementation was administered post-exercise alongside lunch, which may have attenuated potential acute ergogenic effects during training; future studies should systematically compare pre- versus post-exercise supplementation timing to optimize potential performance benefits. Fifth, although resting blood pressure and heart rhythm were assessed prior to each session, the absence of electrocardiographic (ECG) monitoring limits comprehensive cardiovascular safety evaluation; therefore, future trials should incorporate standardized ECG-based screening to strengthen cardiac risk stratification, particularly in older populations. Finally, the absence of a HIIT-only control group limits the ability to disentangle the independent effects of CoQ10 supplementation from those induced by training alone.

## 6. Conclusions

In conclusion, CoQ10 supplementation combined with HIIT was associated with greater improvements in lower-body strength and power in older adults compared with HIIT alone. These effects may be related to improvements in mitochondrial function and antioxidant capacity, potentially contributing to reduced muscular fatigue. However, due to the absence of baseline plasma CoQ10 measurements, causal attribution to supplementation should be interpreted with caution. In contrast, no additional benefits were observed for balance, gait, or aerobic endurance beyond those induced by HIIT alone.

Overall, these findings are consistent with the potential role of CoQ10 as an adjunct to exercise programs targeting strength-related functional outcomes in older adults, rather than definitive evidence of efficacy. Future research should incorporate baseline and post-intervention CoQ10 measurements, explore optimal dosing and timing, interactions with other nutritional strategies, and the long-term effects on physical function and body composition.

## Figures and Tables

**Table 1:** Baseline characteristics of the participants.

Variable	HIIT + Q10	HIIT + Placebo
Anthropometry and body composition		
Age (years)	69 ± 4	70 ± 4
Sex (n)	11 Males, 8 Females	11 Males, 8 Females
Body mass (kg)	70.6 ± 8.8	70.8 ± 9.1
Height (cm)	162.7 ± 10.2	161.9 ± 8
BMI (kg·m^−2^)	26.9 ± 4.9	27.2 ± 4.1
SMM (kg)	24.6 ± 4.25	24.5 ± 4.40
BF (kg)	25.3 ± 9.23	25.8 ± 7.85
Physical function		
5XSST (s)	13.8 ± 3.2	14.3 ± 2.9
30CST (r)	12.1 ± 1.5	12.05 ± 1.9
HG (R;Ib)	26.8 ± 6.7	26.2 ± 6.4
HG (L;Ib)	27.1 ± 5.8	24.6 ± 5.1
TUG (s)	9.2 ± 1.5	9.3 ± 1.8
25FW (s)	6.3 ± 1.3	6.9 ± 1.2
SLS (s)	11.4 ± 7.6	12.2 ± 6.9
6MWT (m)	470.7 ± 60.5	455.3 ± 38.3

Abbreviations: BMI: Body Mass Index; 5XSST: 5-Times Sit-to-Stand Test; 30CST: 30-Second Chair Stand Test; HG (R): Handgrip (Right Hand); HG (L): Handgrip (Left Hand); TUG: Timed Up and Go Test; 25FW: 25-Foot Walk Test; SLS: Single-Leg Stance Test; 6MWT: 6-Minute Walk Test.

**Table 2:** Adherence and fidelity to the intervention across the study period.

Outcome Measure	HIIT + CoQ10	HIIT + Placebo	*p*-Value
Supplement Adherence Rate (%)	98.11 ± 1.74	97.73 ± 2.05	0.549

**Table 3:** Comparison of functional physical performance improvements between the HIIT + CoQ10 and HIIT+ Placebo groups over 8 weeks.

Variable	HIIT + CoQ10 Mean (Confidence Interval 95%)	HIIT + P Mean (Confidence Interval 95%)	*p*-Value
5XSST	−4.26 s (−5.73 to −2.79)	−1.40 s (−2.19 to −0.60)	0.020
30CST	4.31 rep (3.46 to 5.16)	1.63 rep (1.17 to 2.09)	0.014
HG(R)	3.26 Ib (1.27 to 5.24)	3.42 Ib (0.78 to 6.05)	0.812
HG(L)	3.52 Ib (0.89 to 6.15)	2.57 Ib (0.48 to 4.67)	0.463
TUG	−2.37 s (−3.00 to −1.74)	−1.20 s (−1.79 to −0.60)	0.134
25FW	−1.45 s (−1.95 to −0.94)	−1.26 s (−1.87 to −0.66)	0.056
SLS	12.92 s (5.78 to 20.06)	18.93 s (10.61 to 27.25)	0.337
6MWT	51.26 m (39.62 to 62.89)	30.31 m (18.92 to 41.70)	0.132

**Table 4:** Nutrition and dietary intake before and after intervention (M ± SD).

	HIIT + CoQ10		HIIT + Placebo	
	Pre	Post	Pre	Post
Energy (Cal/d)	2057.3 ± 129.8	2056 ± 124.5	2071.3 ± 187.4	2071.8 ± 176.5
Carbohydrate (g/d)	209.6 ± 26.2	206.7 ± 19.3	208 ± 23.9	205.4 ± 20.2
Protein (g/d)	55.4 ± 4.1	56 ± 4.4	55.97 ± 6.33	56.6 ± 5.7
Fat (g/d)	45.3 ± 4.5	45.1 ± 5.1	44.8 ± 5.2	44.9 ± 5.2

## Data Availability

The data that support the findings of this study are available from the corresponding author upon reasonable request. Due to privacy and ethical considerations, the data are not publicly available.

## References

[1] Zhou D.-D., Luo M., Huang S.-Y., Saimaiti A., Shang A., Gan R.-Y., Li H.-B. (2021). Effects and Mechanisms of Resveratrol on Aging and Age-Related Diseases. Oxidative Med. Cell. Longev..

[2] Rizzoli R., Reginster J.-Y., Arnal J.-F., Bautmans I., Beaudart C., Bischoff-Ferrari H., Biver E., Boonen S., Brandi M.-L., Chines A. (2013). Quality of Life in Sarcopenia and Frailty. Calcif. Tissue Int..

[3] Tchkonia T., Morbeck D.E., Von Zglinicki T., Van Deursen J., Lustgarten J., Scrable H., Khosla S., Jensen M.D., Kirkland J.L. (2010). Fat tissue, aging, and cellular senescence. Aging Cell.

[4] Baumgartner R.N. (2000). Body Composition in Healthy Aging. Ann. N. Y. Acad. Sci..

[5] Holmes C.J., Racette S.B. (2021). The Utility of Body Composition Assessment in Nutrition and Clinical Practice: An Overview of Current Methodology. J. Nutr..

[6] Li C.-w., Yu K., Shyh-Chang N., Jiang Z., Liu T., Ma S., Luo L., Guang L., Liang K., Ma W. (2022). Pathogenesis of sarcopenia and the relationship with fat mass: Descriptive review. J. Cachexia Sarcopenia.

[7] Ponti F., Santoro A., Mercatelli D., Gasperini C., Conte M., Martucci M., Sangiorgi L., Franceschi C., Bazzocchi A. (2020). Aging and Imaging Assessment of Body Composition: From Fat to Facts. Front. Endocrinol..

[8] Steenman M., Lande G. (2017). Cardiac aging and heart disease in humans. Biophys. Rev..

[9] Amorim J.A., Coppotelli G., Rolo A.P., Palmeira C.M., Ross J.M., Sinclair D.A. (2022). Mitochondrial and metabolic dysfunction in ageing and age-related diseases. Nat. Rev. Endocrinol..

[10] Cunningham C., O’ Sullivan R., Caserotti P., Tully M.A. (2020). Consequences of physical inactivity in older adults: A systematic review of reviews and meta-analyses. Scand. J. Med. Sci. Sports.

[11] Izquierdo M., Merchant R.A., Morley J.E., Anker S.D., Aprahamian I., Arai H., Aubertin-Leheudre M., Bernabei R., Cadore E.L., Cesari M. (2021). International Exercise Recommendations in Older Adults (ICFSR): Expert Consensus Guidelines. J. Nutr. Health Aging.

[12] Bliss E.S., Wong R.H.X., Howe P.R.C., Mills D.E. (2020). Benefits of exercise training on cerebrovascular and cognitive function in ageing. J. Cereb. Blood Flow Metab..

[13] Bao W., Sun Y., Zhang T., Zou L., Wu X., Wang D., Chen Z. (2020). Exercise Programs for Muscle Mass, Muscle Strength and Physical Performance in Older Adults with Sarcopenia: A Systematic Review and Meta-Analysis. Aging Dis..

[14] Moreira J.B.N., Wohlwend M., Wisløff U. (2020). Exercise and cardiac health: Physiological and molecular insights. Nat. Metab..

[15] Casuso R.A., Huertas J.R. (2020). The emerging role of skeletal muscle mitochondrial dynamics in exercise and ageing. Ageing Res. Rev..

[16] Radaelli R., Taaffe D.R., Newton R.U., Galvão D.A., Lopez P. (2021). Exercise effects on muscle quality in older adults: A systematic review and meta-analysis. Sci. Rep..

[17] Papalia G.F., Papalia R., Diaz Balzani L.A., Torre G., Zampogna B., Vasta S., Fossati C., Alifano A.M., Denaro V. (2020). The Effects of Physical Exercise on Balance and Prevention of Falls in Older People: A Systematic Review and Meta-Analysis. J. Clin. Med..

[18] Scoubeau C., Carpentier J., Baudry S., Faoro V., Klass M. (2023). Body composition, cardiorespiratory fitness, and neuromuscular adaptations induced by a home-based whole-body high intensity interval training. J. Exerc. Sci. Fit..

[19] Yang C., Zhang L., Cheng Y., Zhang M., Zhao Y., Zhang T., Dong J., Xing J., Zhen Y., Wang C. (2024). High intensity interval training vs. moderate intensity continuous training on aerobic capacity and functional capacity in patients with heart failure: A systematic review and meta-analysis. Front. Cardiovasc. Med..

[20] Khodadadi F., Bagheri R., Negaresh R., Moradi S., Nordvall M., Camera D.M., Wong A., Suzuki K. (2023). The Effect of High-Intensity Interval Training Type on Body Fat Percentage, Fat and Fat-Free Mass: A Systematic Review and Meta-Analysis of Randomized Clinical Trials. J. Clin. Med..

[21] Alzar-Teruel M., Aibar-Almazán A., Hita-Contreras F., Carcelén-Fraile M.d.C., Martínez-Amat A., Jiménez-García J.D., Fábrega-Cuadros R., Castellote-Caballero Y. (2022). High-intensity interval training among middle-aged and older adults for body composition and muscle strength: A systematic review. Front. Public Health.

[22] Li J., Li Y., Atakan M.M., Kuang J., Hu Y., Bishop D.J., Yan X. (2020). The Molecular Adaptive Responses of Skeletal Muscle to High-Intensity Exercise/Training and Hypoxia. Antioxidants.

[23] Belzunce M.A., Henckel J., Laura A.D., Horga L.M., Hart A.J. (2023). Mid-life cyclists preserve muscle mass and composition: A 3D MRI study. BMC Musculoskelet. Disord..

[24] Bouaziz W., Schmitt E., Kaltenbach G., Geny B., Vogel T. (2015). Health benefits of cycle ergometer training for older adults over 70: A review. Eur. Rev. Aging Phys. Act..

[25] Lovell D.I., Cuneo R., Gass G.C. (2010). Can aerobic training improve muscle strength and power in older men?. J. Aging Phys. Act..

[26] Gielen E., Beckwée D., Delaere A., De Breucker S., Vandewoude M., Bautmans I. (2021). The Sarcopenia Guidelines Development Group of the Belgian Society of Gerontology and Geriatrics (BSGG). Nutritional interventions to improve muscle mass, muscle strength, and physical performance in older people: An umbrella review of systematic reviews and meta-analyses. Nutr. Rev..

[27] Wirth J., Hillesheim E., Brennan L. (2020). The Role of Protein Intake and its Timing on Body Composition and Muscle Function in Healthy Adults: A Systematic Review and Meta-Analysis of Randomized Controlled Trials. Nutr. J..

[28] Wu P.-Y., Huang K.-S., Chen K.-M., Chou C.-P., Tu Y.-K. (2021). Exercise, Nutrition, and Combined Exercise and Nutrition in Older Adults with Sarcopenia: A Systematic Review and Network Meta-analysis. Maturitas.

[29] Fantacone M.L., Lowry M.B., Uesugi S.L., Michels A.J., Choi J., Leonard S.W., Gombart S.K., Gombart J.S., Bobe G., Gombart A.F. (2020). The Effect of a Multivitamin and Mineral Supplement on Immune Function in Healthy Older Adults: A Double-Blind, Randomized, Controlled Trial. J. Nutr..

[30] Giustina A., Bouillon R., Dawson-Hughes B., Ebeling P.R., Lazaretti-Castro M., Lips P., Marcocci C., Bilezikian J.P. (2023). Vitamin D in the older population: A consensus statement. Endocr. J..

[31] Cornish S.M., Cordingley D.M., Shaw K.A., Forbes S.C., Leonhardt T., Bristol A., Candow D.G., Chilibeck P.D. (2022). Effects of Omega-3 Supplementation Alone and Combined with Resistance Exercise on Skeletal Muscle in Older Adults: A Systematic Review and Meta-Analysis. J. Nutr..

[32] Testai L., Martelli A., Flori L., Cicero A.F.G., Colletti A. (2021). Coenzyme Q10: Clinical Applications beyond Cardiovascular Diseases. J. Nutr..

[33] de la Bella-Garzón R., Fernández-Portero C., Alarcón D., Amián J.G., López-Lluch G. (2022). Levels of Plasma Coenzyme Q10 Are Associated with Physical Capacity and Cardiovascular Risk in the Elderly. Antioxidants.

[34] Drobnic F., Lizarraga M.A., Caballero-García A., Cordova A. (2022). Coenzyme Q(10) Supplementation and Its Impact on Exercise and Sport Performance in Humans: A Recovery or a Performance-Enhancing Molecule?. J. Nutr..

[35] Martelli A., Testai L., Colletti A., Cicero A.F.G. (2020). Coenzyme Q(10): Clinical Applications in Cardiovascular Diseases. Antioxidants.

[36] Lu Y., Wiltshire H.D., Baker J.S., Wang Q. (2021). Effects of High Intensity Exercise on Oxidative Stress and Antioxidant Status in Untrained Humans: A Systematic Review. J. Biol..

[37] Malm C., Svensson M., Ekblom B., Sjödin B. (1997). Effects of ubiquinone-10 supplementation and high intensity training on physical performance in humans. Acta Physiol. Scand..

[38] Braun B., Clarkson P.M., Freedson P.S., Kohl R.L. (1991). Effects of Coenzyme Q10 Supplementation on Exercise Performance, VO2max, and Lipid Peroxidation in Trained Cyclists. Int. J. Sport Nutr..

[39] Belardinelli R., Muçaj A., Lacalaprice F., Solenghi M., Seddaiu G., Principi F., Tiano L., Littarru G.P. (2006). Coenzyme Q10 and exercise training in chronic heart failure. Eur. Heart J..

[40] Belviranlı M., Okudan N. (2019). Effect of coenzyme Q10 alone and in combination with exercise training on oxidative stress biomarkers in rats. Int. J. Vitam. Nutr. Res..

[41] Hernández-Camacho J.D., Bernier M., López-Lluch G., Navas P. (2018). Coenzyme Q10 supplementation in aging and disease. Front. Physiol..

[42] Reljic D., Wittmann F., Fischer J.E. (2018). Effects of low-volume high-intensity interval training in a community setting: A pilot study. Eur. J. Appl. Physiol..

[43] Liang W., Wang X., Cheng S., Jiao J., Zhu X., Duan Y. (2024). Effects of High-Intensity Interval Training on the Parameters Related to Physical Fitness and Health of Older Adults: A Systematic Review and Meta-Analysis. Sports Med. Open.

[44] Men J., Zhao C., Xiang C., Zhu G., Yu Z., Wang P., Wu S., Zhang Y., Li Y., Wang L. (2025). Effects of high-intensity interval training on physical morphology, cardiopulmonary function, and metabolic indicators in older adults: A systematic review and meta-analysis. Front. Endocrinol..

[45] Abdali D., Samson S.E., Grover A.K. (2015). How Effective Are Antioxidant Supplements in Obesity and Diabetes?. Med. Princ. Pract..

[46] Talebi S., Pourgharib Shahi M.H., Zeraattalab-Motlagh S., Asoudeh F., Ranjbar M., Hemmati A., Talebi A., Wong A., Mohammadi H. (2024). The effects of coenzyme Q10 supplementation on biomarkers of exercise-induced muscle damage, physical performance, and oxidative stress: A GRADE-assessed systematic review and dose-response meta-analysis of randomized controlled trials. Clin. Nutr. ESPEN.

[47] Tapanya W., Sangkarit N., Amput P., Konsanit S. (2023). Lower extremity muscle strength equation of older adults assessed by Five Time Sit to Stand Test (FTSST). Hong Kong Physiother. J..

[48] Mahato N.K., Davis A., Simon J.E., Clark B.C. (2024). Assessing muscular power in older adults: Evaluating the predictive capacity of the 30-second chair rise test. Front. Aging Neurosci..

[49] Roberts H.C., Denison H.J., Martin H.J., Patel H.P., Syddall H., Cooper C., Sayer A.A. (2011). A review of the measurement of grip strength in clinical and epidemiological studies: Towards a standardised approach. Age Ageing.

[50] Podsiadlo D., Richardson S. (1991). The timed “Up & Go”: A test of basic functional mobility for frail elderly persons. J. Am. Geriatr. Soc..

[51] Tappen R.M., Roach K.E., Buchner D., Barry C., Edelstein J. (1997). Reliability of physical performance measures in nursing home residents with Alzheimer’s disease. J. Gerontol. Ser. A Biol. Sci. Med. Sci..

[52] Vellas B.J., Wayne S.J., Romero L., Baumgartner R.N., Rubenstein L.Z., Garry P.J. (1997). One-leg balance is an important predictor of injurious falls in older persons. J. Am. Geriatr. Soc..

[53] Guyatt G.H., Sullivan M.J., Thompson P.J., Fallen E.L., Pugsley S.O., Taylor D.W., Berman L.B. (1985). The 6-minute walk: A new measure of exercise capacity in patients with chronic heart failure. Can. Med. Assoc. J..

[54] Craig C., Marshall A., Sjostrom M., Bauman A., Lee P., Macfarlane D., Lam T., Stewart S. (2017). International physical activity questionnaire-short form. J. Am. Coll. Health.

[55] Ochiai A., Itagaki S., Kurokawa T., Kobayashi M., Hirano T., Iseki K. (2007). Improvement in intestinal coenzyme q10 absorption by food intake. Yakugaku Zasshi.

[56] Li Z., Kopec R.E. (2024). CoQ10 bioaccessibility and Caco-2 cell uptake improved with novel medium chain triglyceride encapsulation. Food Funct..

[57] Estevez M.B., Casaux M.L., Fraga M., Faccio R., Alborés S. (2021). Biogenic Silver Nanoparticles as a Strategy in the Fight Against Multi-Resistant Salmonella enterica Isolated From Dairy Calves. Front. Bioeng. Biotechnol..

[58] Maes M., Mihaylova I., Kubera M., Uytterhoeven M., Vrydags N., Bosmans E. (2009). Coenzyme Q10 deficiency in myalgic encephalomyelitis/chronic fatigue syndrome (ME/CFS) is related to fatigue, autonomic and neurocognitive symptoms and is another risk factor explaining the early mortality in ME/CFS due to cardiovascular disorder. Neuro Endocrinol. Lett..

[59] Cooke M., Iosia M., Buford T., Shelmadine B., Hudson G., Kerksick C., Rasmussen C., Greenwood M., Leutholtz B., Willoughby D. (2008). Effects of acute and 14-day coenzyme Q10 supplementation on exercise performance in both trained and untrained individuals. J. Int. Soc. Sports Nutr..

[60] Chen H.-C., Huang C.-C., Lin T.-J., Hsu M.-C., Hsu Y.-J. (2019). Ubiquinol Supplementation Alters Exercise Induced Fatigue by Increasing Lipid Utilization in Mice. Nutrients.

[61] Grevendonk L., Connell N.J., McCrum C., Fealy C.E., Bilet L., Bruls Y.M.H., Mevenkamp J., Schrauwen-Hinderling V.B., Jörgensen J.A., Moonen-Kornips E. (2021). Impact of aging and exercise on skeletal muscle mitochondrial capacity, energy metabolism, and physical function. Nat. Commun..

[62] Chen M., Wang Y., Deng S., Lian Z., Yu K. (2022). Skeletal muscle oxidative stress and inflammation in aging: Focus on antioxidant and anti-inflammatory therapy. Front. Cell Dev. Biol..

[63] Pala R., Orhan C., Tuzcu M., Sahin N., Ali S., Cinar V., Atalay M., Sahin K. (2016). Coenzyme Q10 Supplementation Modulates NFκB and Nrf2 Pathways in Exercise Training. J. Sports Sci. Med..

[64] Sangsefidi Z.S., Yaghoubi F., Hajiahmadi S., Hosseinzadeh M. (2020). The effect of coenzyme Q10 supplementation on oxidative stress: A systematic review and meta-analysis of randomized controlled clinical trials. Food Sci. Nutr..

[65] Supruniuk E., Górski J., Chabowski A. (2023). Endogenous and Exogenous Antioxidants in Skeletal Muscle Fatigue Development during Exercise. Antioxidants.

[66] Powers S.K., Goldstein E., Schrager M., Ji L.L. (2023). Exercise Training and Skeletal Muscle Antioxidant Enzymes: An Update. Antioxidants.

[67] Zhu Y., Zhou X., Zhu A., Xiong S., Xie J., Bai Z. (2023). Advances in exercise to alleviate sarcopenia in older adults by improving mitochondrial dysfunction. Front. Physiol..

[68] Andreani C., Bartolacci C., Guescini M., Battistelli M., Stocchi V., Orlando F., Provinciali M., Amici A., Marchini C., Tiano L. (2018). Combination of Coenzyme Q10 Intake and Moderate Physical Activity Counteracts Mitochondrial Dysfunctions in a SAMP8 Mouse Model. Oxidative Med. Cell. Longev..

[69] Betz M.W., Hendriks F.K., Houben A.J.H.M., van den Eynde M.D.G., Verdijk L.B., van Loon L.J.C., Snijders T. (2023). Type II Muscle Fiber Capillarization Is an Important Determinant of Post-Exercise Microvascular Perfusion in Older Adults. Gerontology.

[70] Hung C.-H., Su C.-H., Wang D. (2025). The Role of High-Intensity Interval Training (HIIT) in Neuromuscular Adaptations: Implications for Strength and Power Development—A Review. Life.

[71] Wiens L., Losciale J.M., Fliss M.D., Abercrombie M.J., Darabi D., Li J., Barclay R., Mitchell C.J. (2025). Does High-Intensity Interval Training Increase Muscle Strength, Muscle Mass, and Muscle Endurance? A Systematic Review and Meta-Analysis. Sports.

[72] Wu Z.-J., Wang Z.-Y., Gao H.-E., Zhou X.-F., Li F.-H. (2021). Impact of high-intensity interval training on cardiorespiratory fitness, body composition, physical fitness, and metabolic parameters in older adults: A meta-analysis of randomized controlled trials. Exp. Gerontol..

[73] Amaro-Gahete F.J., De-la-O A., Jurado-Fasoli L., Ruiz J.R., Castillo M.J., Gutiérrez Á. (2019). Effects of different exercise training programs on body composition: A randomized control trial. Scand. J. Med. Sci. Sports.

[74] Lira V.A., Benton C.R., Yan Z., Bonen A. (2010). PGC-1alpha regulation by exercise training and its influences on muscle function and insulin sensitivity. Am. J. Physiol. Endocrinol. Metab..

[75] Gökbel H., Gül I., Belviranl M., Okudan N. (2010). The effects of coenzyme Q10 supplementation on performance during repeated bouts of supramaximal exercise in sedentary men. J. Strength Cond. Res..

[76] Fogacci F., Giovannini M., Tocci G., Imbalzano E., Borghi C., Cicero A.F.G. (2024). Effect of Coenzyme Q(10) on Physical Performance in Older Adults with Statin-Associated Asthenia: A Double-Blind, Randomized, Placebo-Controlled Clinical Trial. J. Clin. Med..

[77] Fernandes M.S.S., Fidelis D., Aidar F.J., Badicu G., Greco G., Cataldi S., Santos G.C.J., de Souza R.F., Ardigò L.P. (2023). Coenzyme Q10 Supplementation in Athletes: A Systematic Review. Nutrients.

